# Effects of highly active antiretroviral therapy initiation on epigenomic DNA methylation in persons living with HIV

**DOI:** 10.3389/fbinf.2024.1357889

**Published:** 2024-05-24

**Authors:** Joshua Zhang, Mary E. Sehl, Roger Shih, Elizabeth Crabb Breen, Fengxue Li, Ake T. Lu, Jay H. Bream, Priya Duggal, Jeremy Martinson, Steven M. Wolinsky, Otoniel Martinez-Maza, Christina M. Ramirez, Steve Horvath, Beth D. Jamieson

**Affiliations:** ^1^ Department of Human Genetics, David Geffen School of Medicine at UCLA, University of California Los Angeles, Los Angeles, CA, United States; ^2^ Division of Hematology-Oncology, Department of Medicine, David Geffen School of Medicine at UCLA, University of California Los Angeles, Los Angeles, CA, United States; ^3^ Department of Computational Medicine, David Geffen School of Medicine at UCLA, University of California Los Angeles, Los Angeles, CA, United States; ^4^ Department of Psychiatry and Biobehavioral Sciences, Cousins Center for Psychoneuroimmunology, David Geffen School of Medicine at UCLA, University of California Los Angeles, Los Angeles, CA, United States; ^5^ Department of Biostatistics, Fielding School of Public Health, University of California Los Angeles, Los Angeles, CA, United States; ^6^ Altos Labs, San Diego Institute of Science, San Diego, CA, United States; ^7^ Department of Molecular Microbiology and Immunology, Johns Hopkins Bloomberg School of Public Health, Immunology Training Program, Johns Hopkins School of Medicine, Baltimore, MD, United States; ^8^ Department of Epidemiology, Johns Hopkins Bloomberg School of Public Health, Baltimore, MD, United States; ^9^ Department of Infectious Diseases and Microbiology, Graduate School of Public Health, University of Pittsburgh, Pittsburgh, PA, United States; ^10^ Department of Medicine, Northwestern University Feinberg School of Medicine, Chicago, IL, United States; ^11^ Departments of Obstetrics and Gynecology and Microbiology, Immunology and Molecular Genetics, David Geffen School of Medicine at UCLA, University of California, Los Angeles, CA, United States

**Keywords:** DNA methylation, epigenetics, EWAS, bioinformatics, HIV, antiretroviral therapy

## Abstract

**Introduction:** Highly active antiretroviral therapy (HAART) helps improve some measures of accelerated epigenetic aging in persons living with HIV (PLWH), but its overall impact on the epigenome is not fully understood.

**Methods:** In this study, we analyzed the DNA methylation profiles of PLWH (*n* = 187) shortly before and approximately 2–3 years after they started HAART, as well as matched seronegative (SN) controls (*n* = 187), taken at two time intervals. Our aim was to identify specific CpGs and biologic pathways associated with HIV infection and initiation of HAART. Additionally, we attempted to identify epigenetic changes associated with HAART initiation that were independent of HIV-associated changes, using matched HIV seronegative (SN) controls (matched on age, hepatitis C status, and interval between visits) to identify CpGs that did not differ between PLWH and SN pre-HAART but were significantly associated with HAART initiation while being unrelated to HIV viral load. Epigenome-wide association studies (EWAS) on >850,000 CpG sites were performed using pre- and post-HAART samples from PLWH. The results were then annotated using the Genomic Regions Enrichment of Annotations Tool (GREAT).

**Results:** When only pre- and post-HAART visits in PLWH were compared, gene ontologies related to immune function and diseases related to immune function were significant, though with less significance for PLWH with detectable HIV viral loads (>50 copies/mL) at the post-HAART visit. To specifically elucidate the effects of HAART separately from HIV-induced methylation changes, we performed EWAS of HAART while also controlling for HIV viral load, and found gene ontologies associated with transplant rejection, transplant-related diseases, and other immunologic signatures. Additionally, we performed a more focused analysis that examined CpGs reaching genome-wide significance (*p* < 1 × 10^−7^) from the viral load-controlled EWAS that did not differ between all PLWH and matched SN controls pre-HAART. These CpGs were found to be near genes that play a role in retroviral drug metabolism, diffuse large B cell lymphoma proliferation, and gastric cancer metastasis.

**Discussion:** Overall, this study provides insight into potential biological functions associated with DNA methylation changes induced by HAART initiation in persons living with HIV.

## 1 Introduction

Highly active antiretroviral therapy (HART) has been demonstrated to reduce viral load, improve immune function, reduce morbidity and mortality, and improve quality of life in persons living with HIV (PLWH) (Thompson et al., 2012). However, even when adequate viral suppression is achieved, PLWH have an increased risk of cardiovascular disease, chronic kidney disease, osteopenia, and type 2 diabetes mellitus (Lerner et al., 2020). The molecular mechanisms underlying the increased risk of these coexisting illnesses in PLWH remain poorly understood. Epigenome-wide association studies (EWAS) have demonstrated differentially methylated CpGs in PLWH compared with age-matched control participants in genes related to antiviral immunity and cytokine response (NLRC5) and in the HIV 5’ long terminal repeat promoter, which is essential for replication and genome packaging (Blazkova et al., 2009; Blazkova et al., 2012; Zhang et al., 2016; Shiau et al., 2018; Titanji et al., 2022). Further EWAS investigations have identified differentially methylated groups of CpGs associated with airflow obstruction (Hernandez Cordero et al., 2021), declining estimated glomerular filtration rate (Chen et al., 2020), cognitive impairment (Corley et al., 2016), accelerated epigenetic aging (Rickabaugh et al., 2015; Breen et al., 2022; Sehl et al., 2024), and mortality (Shu et al., 2021) in PLWH. It is important to disentangle the effects of HIV versus its treatment on the differential methylation patterns observed in PLWH. Longitudinal studies are needed to examine which of these methylation patterns are affected by the initiation of HAART.

In the current study, we examine EWAS using peripheral blood mononuclear cells (PBMC) from 200 PLWH within 1.5 years prior to and 2–3 years after HAART initiation, and in HIV seronegative control participants (SN) matched on age, hepatitis C virus, and time intervals. Using a linear mixed-effects model, we identify differentially methylated groups of CpGs that are associated with the initiation of HAART, while controlling for chronologic age, HIV status, and viral load. We further stratify PLWH into two groups based on viral load suppression, and identify HAART-related CpGs within each group. Finally, we searched for enrichment terms to identify functional characteristics of HAART-induced changes in methylation using the Genomic Regions Enrichment of Annotations Tool (GREAT). Our study represents the first large longitudinal study examining differential methylation patterns in PLWH and matched SN participants using models accounting for advancing age and viral suppression.

## 2 Materials and methods

### 2.1 Participants and samples

We used archived viably cryopreserved peripheral blood mononuclear cells from the Multicenter AIDS Cohort Study (MACS), now the MACS/MACS WIHS Combined Cohort Study (MWCCS). As described in Sehl et al., 2024, We selected samples from 200 persons living with HIV (PLWH) at a pre-HAART (Visit 1) within 1.5 years prior to HAART initiation, and a post-HAART visit (Visit 2; 400 unique PLWH samples total) 2.5–3 years post HAART initiation. We then selected 199 persistently HIV seronegative (SN) men who were matched to PLWH on chronologic age and Hepatitis C virus status. Samples from these SN controls were selected from visits temporally comparable to their matched PLWH sample. One SN participant served as a control for two different PLWH due to the requirement for matched Hepatitis C virus status. Four separate samples were therefore selected from this person (400 unique SN samples total; 800 samples overall). CMV status was not included due to the high prevalence of MACS participants showing seropositivity for cytomegalovirus (CMV, 97%–100%) prior to HIV infection (Breen et al., 2022), consistent with other reports of CMV prevalence in men-who-have-sex-with-men (Drew et al., 1981; Nerurkar et al., 1987).

### 2.2 Definition and initiation of HAART

Initiation of HAART was determined by the MWCCS. Briefly, use of antiretroviral therapy medications was initially self reported then confirmed by medical record reviews. The initiation of HAART is defined as halfway between the last visit at which no HAART use was reported and the first visit at which HAART use was reported. The 2008 definition of HAART was used for this study and was composed of at least 3 antiretroviral therapies, including: 2 nucleoside reverse transcriptase inhibitors (NRTIs) plus either an unboosted protease inhibitor (PI) or a boosted PI or a non-nucleoside reverse transcriptase inhibitor (NNRTI) (Castillo-Mancilla et al., 2020).

### 2.3 Genomic DNA isolation, quantification and methylation arrays

Genomic DNA was isolated from a pellet of approximately one million thawed and washed peripheral blood mononuclear cells. Samples were stored in a −80°C freezer. DNA extraction was performed as described elsewhere (Breen et al., 2022). The DNA was quantified using a NanoDrop One (ThermoFisher) using the dsDNA setting and automatic measurements generated from 220 to 340 nm wavelengths. Genomic DNA samples were then stored in −80°C freezers until plated for methylation analysis using the Illumina Infinium MethylationEPIC BeadChip (Illumina, San Diego, CA). Samples were plated using blinded matched sets of genomic DNA samples and each set contained samples from matched PLWH and SN participants at all visits. Plates were assayed by the UCLA Neuroscience Genomics Core (https://www.semel.ucla.edu/ungc), as previously described (Breen et al., 2022). DNA methylation levels (beta values) were determined by calculating the intensity of the methylated and unmethylated sites as the ratio of fluorescent signals, yielding beta values that range from 0 (completely un-methylated) to 1 (completely methylated). Quantile normalization was applied to the raw data, to detect and remove outliers, and to make data comparable to the training data of the epigenetic clocks and consistent with previous analyses (Sehl et al., 2020).

### 2.4 Methylation data and final analytical sample

We performed single sample noob normalization using the minfi R package (Aryee et al., 2014) on the raw methylation data for all 800 samples. Non-CG probes, SNP probes, and sex chromosome probes were removed prior to further analysis. Hierarchical clustering was used to identify misclustered samples, i.e., paired samples (Visits 1 and 2) labeled as belonging to the same individual but were identified from the methylation data as having a high probability of belonging to two different individuals (*n* = 12 pairs). The 12 misclustered pairs of samples, as well as the 12 pairs of Visit 1 and 2 samples matched to the misclustered samples (total of 48 samples), were excluded from the analyses (*n* = 752 total remaining samples). One PLWH did not have VL data available at visit 2, and as a result was excluded at both visits, along with the matched SN at both visits, for a final *n* = 748 samples (187 PLWH matched with 187 SN controls at both visits) in the analytical sample. PLWH were then split into two groups defined as having Undetected (≤50 copies/mL, *n* = 120) or Detected (>50 copies/mL, *n* = 67) HIV plasma viral load (VL) at their post-HAART visit (visit 2). Overall mean methylation values at each visit were analyzed by averaging all of the CpG methylation beta values across the relevant samples, in each of the two PLWH groups, as well as for each PLWH group’s respective matched SN controls. The significance values were obtained from Welch’s t-tests conducted between each PLWH group and their matched SN controls.

### 2.5 Epigenome-wide association studies (EWAS)

In initial EWAS analyses, we examined linear mixed effects models with only samples from PLWH. In these analyses, each CpG methylation beta value was regressed upon chronologic age and HAART initiation status (coded as 0 for no HAART ever, up to and including the visit; coded as 1 for those who initiated HAART prior to the visit), with a random intercept for intra-subject correlation. We then performed similar linear mixed effects model analyses within two subgroups: the Undetected (≤50 copies/mL) and Detected (>50 copies/mL) subgroups of the PLWH group. Finally, we examined EWAS within PLWH with adjustment for viral load, where methylation beta values at each CpG were regressed on chronologic age, HAART initiation status, log10-transformed HIV viral load (copies per mL), and a random intercept for intra-subject correlation.

### 2.6 Genomic regions enrichment of annotations tool (GREAT)

Using the rGREAT R package (McLean et al., 2010; R Core Team, 2020; Gu and Hübschmann, 2022), we identified functional annotations of the most significant CpGs related to HAART initiation treatment. We used the top 4,000 most significant CpGs from each EWAS, taking the top 2000 positively correlated (increased methylation with treatment) CpGs and the top 2000 negatively correlated (decreased methylation with treatment) CpGs. For the background set, we used the set of CpGs on the methylation array. We employed the default settings: 5.0 kb upstream and 1.0 kb downstream for Proximal, 50 kb for Distal.

### 2.7 Two one-sided tests (TOST)

We used the TOSTER R package (Lakens, 2017; Caldwell, 2022) to perform equivalence tests on CpG methylation values from pre-HAART initiation (PLWH) and comparable (SN) samples (visit 1), comparing PLWH cases to matched SN controls to find CpGs that were equivalent. The TOST method uses the presence of a true effect that is equal to or greater than the upper bound or equal to or less than the lower bound as the null hypothesis, with the alternative hypothesis being that the effect lies within the upper and lower bounds. CpGs that rejected the null equivalence hypothesis and were also unable to reject the null significance hypothesis of an effect equal to zero were considered to be equivalent. We selected an upper bound and lower bound of a ±0.00553 difference in mean methylation value, determined by taking the median absolute difference between the mean methylation values across all CpGs of PLWH and SN controls at visit 1. The most significant CpGs that rejected the null equivalence hypothesis and were unable to reject the null significance hypothesis of zero effect were then intersected with CpGs that were significantly affected by HAART initiation while controlling for viral load.

## 3 Results

### 3.1 Characteristics of study samples

Participants of this EWAS study are 187 matched pairs of PLWH and SN controls (374 total) who were part of the Multicenter AIDS Cohort Study (MACS). MACS is an ongoing study of men who have sex with men, some of whom have been infected with human immunodeficiency virus (HIV) and others who have remained persistently HIV uninfected (Kaslow et al., 1987). MACS is now part of the MACS/WIHS Combined Cohort Study (MWCCS). The participants whose samples were selected were consistent with the demographics of the MACS overall; the majority were white and had one or more year(s) of college education; additional characteristics in PLWH and SN at each visit are shown in Table 1. In accordance with the study design and matching criteria, both PLWH and SN participants had a mean time of 2–3 years between the two visits evaluated in these analyses.

**TABLE 1:** PLWH and SN sample characteristics.

	PLWH		SN	
Participants, *n*	187		187	
Non-white Race, *n* (%)	26 (13.9)		69 (36.9)	
≥1 year College Education, *n* (%)	167 (89.3)		154 (82.4)	
Visit 1 to Visit 2 in years, mean (SD)	2.9 (0.4)		2.7 (0.8)	

	PLWH		SN	
	Visit 1	Visit 2	Visit 1	Visit 2
Age in years, mean (SD), [range]	44.1 (7.9) [24.1–72.4] *n* = 187	46.9 (8) [27–75.4] *n* = 187	43.9 (8.1) [22.3–71.1] *n* = 187	46.6 (8.2) [24.6–73.8] *n* = 187
Hepatitis C Virus RNA-positive, *n* (%)	8 (4.3) *n* = 187	10 (5.3) *n* = 187	8 (4.3) *n* = 187	10 (5.3) *n* = 187
Hepatitis B Virus surface antigen-positive, *n* (%)	4 (2.1) *n* = 186	5 (2.7) *n* = 186	3 (1.6) *n* = 187	3 (1.6) *n* = 187
Body mass index in kg/m^2^, mean (SD)	25.6 (4.1) *n* = 175	25.5 (3.8) *n* = 174	26.1 (4.9) *n* = 179	26.6 (4.9) *n* = 178
Smoking in cumulative pack years, mean (SD)	13.6 (20) *n* = 183	14.3 (20) *n* = 184	10.5 (15) *n* = 183	11.1 (16) *n* = 183
Absolute CD4 T cell count in cells/mm^3^, mean (SD)	421 (235) *n* = 186	562 (281) *n* = 186	993 (324) *n* = 181	971 (299) *n* = 183
Plasma HIV viral load in copies/mL, mean (SD), median, [range]	67,684 (137,340)	8,991 (58,851)	n/a	n/a
	23,037 [50–948,000] *n* = 177	50 [20–775,439] *n* = 187		
Estimated time since HIV infection in years, mean (SD)	9.2 (4.2) *n* = 162	12.1 (4.4) *n* = 162	n/a	n/a

### 3.2 Epigenome-wide association studies (EWAS) of HAART initiation in PLWH

We used linear mixed-effect regression models to identify CpGs associated with HAART initiation in PLWH. We adjusted for chronologic age in all models and added the participant identifier as the random effect. The data were divided into two groups based on viral load (VL) at Visit 2: Undetected (VL ≤ 50 copies/mL, *n* = 120) and Detected (>50 copies/mL, *n* = 67). EWAS analyses were performed separately on these two groups. We used a significance threshold of *p*-value < 1 × 10^−7^ to account for multiple testing.

**FIGURE 1:**
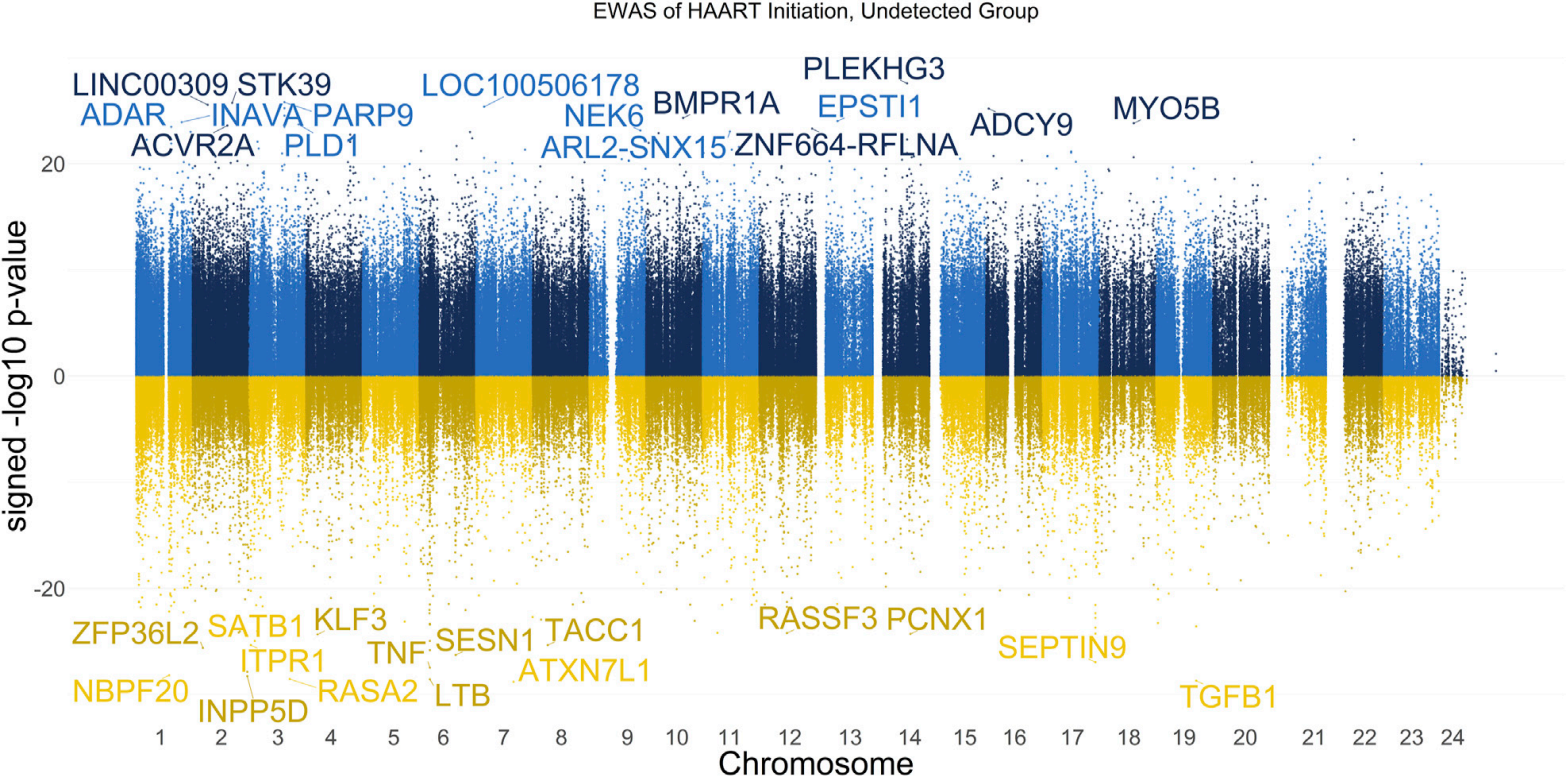
Manhattan Plot of EWAS over the course of HAART initiation in Undetected PLWH Group. Plotted are the HAART-related CpGs in the Undetected PLWH group (plasma HIV VL < or = 50 copies/mL at the post-HAART visit, *n* = 120). Top CpGs are labeled with the names of proximal genes, with the *x*-axis representing the location of the CpG on the chromosome and the *y*-axis representing the signed -log10 *p*-value. The positive y-values (signed -log10 *p*-value) are CpGs that gain methylation with HAART initiation (blue), while the negative y-values are CpGs that lose methylation with HAART initiation (yellow).

The results of our EWAS that examines CpGs associated with HAART initiation within the Undetected group are shown in Figure 1. A total of 62,670 CpGs passed the adjusted significance threshold of *p*-value < 1 × 10^−7^ (Supplementary Table S1). Out of the 4,998 significant CpGs demonstrating loss of methylation from pre-to post-HAART, the most significant CpGs are located in the intron of *ATXN7L1* (cg25652701, *p*-value = 1.53 × 10^−29^), the exon of *TGFB1* (cg27540367, *p*-value = 2.01 × 10^−29^), the exon of *LTB* (cg19279042, *p*-value = 2.68 × 10^−29^), the promoter of *RASA2* (cg09189780, *p*-value = 2.80 × 10^−29^), and the intron of *INPP5D* (cg20416013, *p*-value = 5.26 × 10^−29^). Out of the 57,672 significant CpGs demonstrating gain of methylation from pre-to post-HAART, the most significant CpGs are located in the intron of *PLEKHG3* (cg07315815, *p*-value = 2.69 × 10^−28^), the intron of *PARP9* (cg07815522, *p*-value = 1.64 × 10^−26^), the intron of *STK39* (cg22585786, *p*-value = 1.87 × 10^−26^), the intergenic region upstream of *LINC00309* (cg20501754, *p*-value = 2.83 × 10^−26^), and the intergenic region downstream of *LOC100506178* (cg03695678, *p*-value = 4.29 × 10^−26^).

**FIGURE 2:**
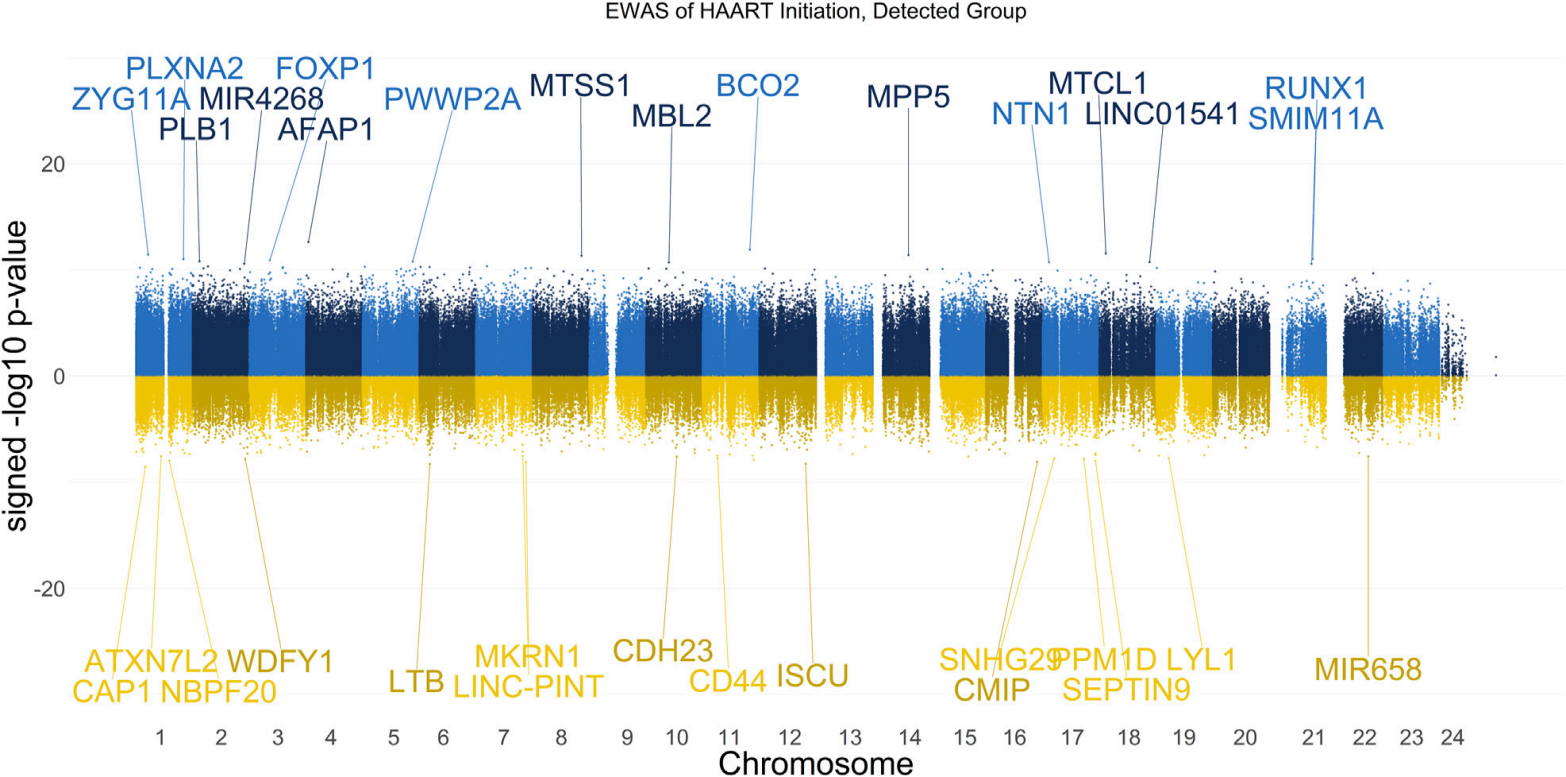
Manhattan Plot of EWAS over the course of HAART Initiation in Detected PLWH Group. Plotted are the HAART-related CpGs in the Detected (plasma HIV VL > 50 copies/mL at the post-HAART visit, *n* = 67) PLWH group. Top CpGs are labeled with proximal genes, the *x*-axis represents the location of the CpG on the chromosome, and the *y*-axis represents the signed -log10 *p*-value. The positive y-values (signed -log10 *p*-value) are CpGs that gain methylation with HAART initiation (blue), while the negative y-values are CpGs that lose methylation with HAART initiation (gold).

The results of our EWAS that examines CpGs associated with HAART initiation within the Detected group are shown in Figure 2. A total of 2,056 CpGs passed the adjusted significance threshold of *p*-value < 1 × 10^−7^ (Supplementary Table S2). Out of the 44 significant CpGs demonstrating loss of methylation from pre-to post-HAART, the most significant CpGs are located in the promoter of *CAP1* (cg24705125, *p*-value = 2.81 × 10^−9^), the exon of *LTB* (cg19279042, *p*-value = 5.14 × 10^−9^), the intron of *ISCU* (cg22277972, *p*-value = 5.54 × 10^−9^), the promoter of *MKRN1* (cg15507271, *p*-value = 7.62 × 10^−9^), and the promoter of *CMIP* (cg27656614, *p*-value = 8.20 × 10^−9^). Out of the 2,012 significant CpGs demonstrating gain of methylation from pre-to post-HAART, the most significant CpGs are located in the intron of *AFAP1* (cg05370755, *p*-value = 2.34 × 10^−13^), the promoter of *BCO2* (cg26581504, *p*-value = 1.25 × 10^−12^), the exon of *MTCL1* (cg16874861, *p*-value = 2.77 × 10^−12^), the intron of *ZYG11A* (cg24497320, *p*-value = 3.75 × 10^−12^), and the intron of *MPP5* (cg13014846, *p*-value = 4.06 × 10^−12^).

**FIGURE 3:**
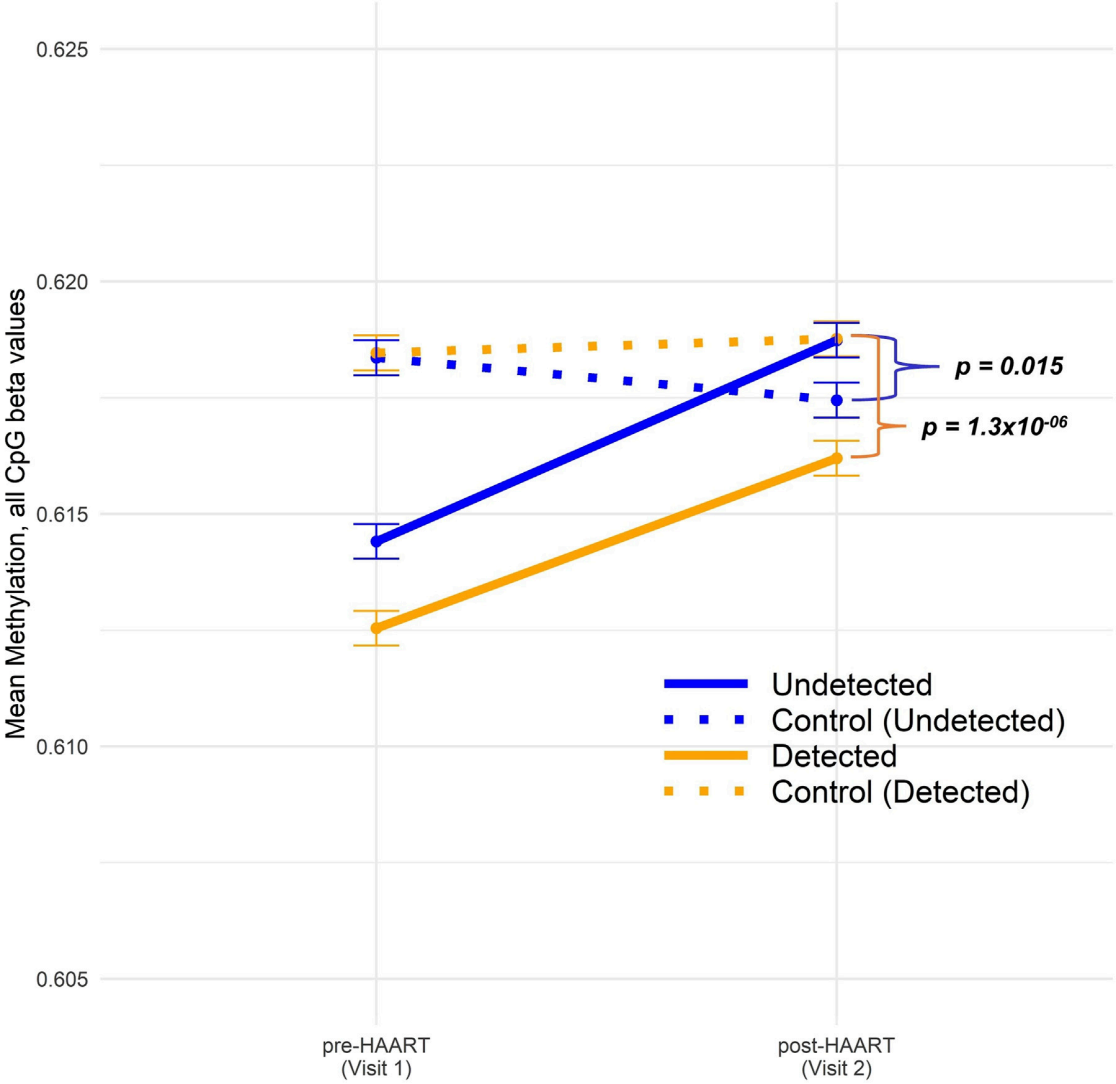
Comparison of epigenome-wide CpG methylation in PLWH pre and post-HAART initiation to SN controls. The CpG methylation data across all sites were split into four groups: Undetected (solid blue) and Detected (solid orange) PLWH, as described in the text, and SN controls for Undetected (dotted blue) and Detected (dotted orange). “Control (Undetected)” refers to Undetected PLWH matched to SN controls and “Control (Detected)” refers to Detected PLWH matched to SN controls. Visit 1/Pre-HAART and Visit 2/post-HAART mean methylation values were plotted for each group along with error bars representing ±1 SE. Welch’s t-tests were conducted between undetected PLWH and their matched SN controls (*p*-value = 0.015) as well as between detected PLWH and their matched SN controls (*p*-value 1.3 × 10^−6^).

Figure 3 shows our analysis of the mean changes in global epigenome-wide methylation in CpGs when comparing PLWH with SN participants over the course of HAART initiation (or comparable time intervals for the SN participants). The overall global methylation levels in PLWH trended upward toward values similar to the matched SN controls after HAART initiation, but remained significantly different (Figure 3). Within the Detected group, the difference between global methylation levels in PLWH and SN was highly significant at Visit 2 (post-HAART, *p* = 1.3 × 10^−6^; Welch’s *t*-test comparing mean CpG values in each PLWH group to their matched SN controls), while within the Undetected group, the mean difference in global methylation between PLWH and SN and the significance (*p* = 0.015) were diminished.

### 3.3 GREAT functional annotation of top HAART initiation-related CpGs

We used GREAT to perform functional annotation using the top 4000 most significant HAART initiation-related CpGs from each EWAS, which consists of the top 2000 positively correlated CpGs (increased methylation) and the top 2000 negatively correlated CpGs (decreased methylation). Each set of positively and negatively correlated CpGs were separately annotated using GREAT and the resulting annotations were subjected to a cutoff of an FDR-adjusted hypergeometric *p*-value of < 0.05 and a non-FDR-adjusted hypergeometric *p*-value of < 0.001.

**FIGURE 4:**
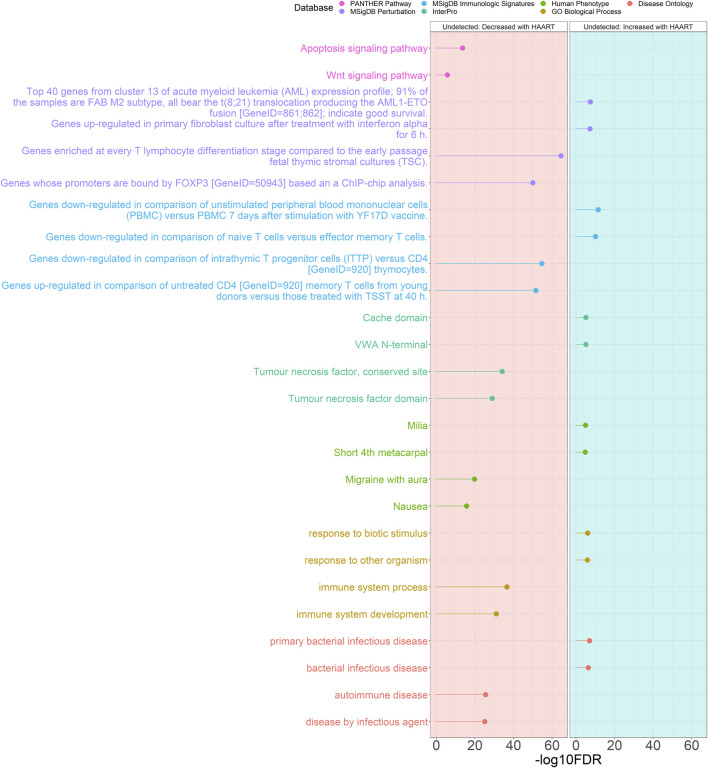
Genomic Regions Enrichment of Annotations Tool for Undetected PLWH groups. GREAT results for HAART-related CpGs in Undetected PLWH groups. The top 2000 positively-correlated CpGs (increased methylation) and the top 2000 negatively-correlated CpGs (decreased methylation) were taken from the EWAS of HAART initiation to use for the GREAT analysis. The graph with the red background shows the enrichment of the set of negatively-correlated CpGs, while the blue background is the enrichment of the set of positively-correlated CpGs. The significant annotations (*y*-axis) are plotted against the -log10 FDR-adjusted hypergeometric *p*-value (*x*-axis). The ontology databases are grouped by color.

The results of the GREAT functional annotation for the Undetected PLWH group are shown in Figure 4. A total of 2,055 annotations passed the significance thresholds (Supplementary Table S3). Out of the 1,618 significant annotations enriched in genes closest to CpGs demonstrating loss of methylation, the most significant annotations were “autoimmune disease” (FDR = 2.82 × 10^−26^), “immune system process” (FDR = 2.94 × 10^−37^), “migraine with aura” (FDR = 1.71 × 10^−20^), “tumour necrosis factor, conserved site” (FDR = 7.79 × 10^−35^), “genes downregulated in comparison of intrathymic T progenitor cells (ITTP) versus CD4 [GeneID = 920] thymocytes” (FDR = 1.88 × 10^−55^), “genes enriched at every T lymphocyte differentiation stage compared to the early passage fetal thymic stromal cultures (TSC)” (FDR = 2.40 × 10^−65^), and “apoptosis signaling pathway” (FDR = 2.83 × 10^−14^). Out of the 437 significant annotations enriched in genes closest to CpGs demonstrating gain of methylation, the most significant annotations were “primary bacterial infectious disease” (FDR = 1.05 × 10^−7^), “response to biotic stimulus” (FDR = 9.41 × 10^−7^), “milia” (FDR = 1.28 × 10^−5^), “VWA N-terminal” (FDR = 7.33 × 10^−6^), “genes downregulated in comparison of unstimulated peripheral blood mononuclear cells (PBMC) versus PBMC 7 days after stimulation with YF17D vaccine” (FDR = 3.17 × 10^−12^), and “top 40 genes from cluster 13 of acute myeloid leukemia (AML) expression profile; 91% of the samples are FAB M2 subtype, all bear the t (8; 21) translocation producing the AML1-ETO fusion [GeneID = 861; 862]; indicate good survival” (FDR = 3.43 × 10^−8^).

**FIGURE 5:**
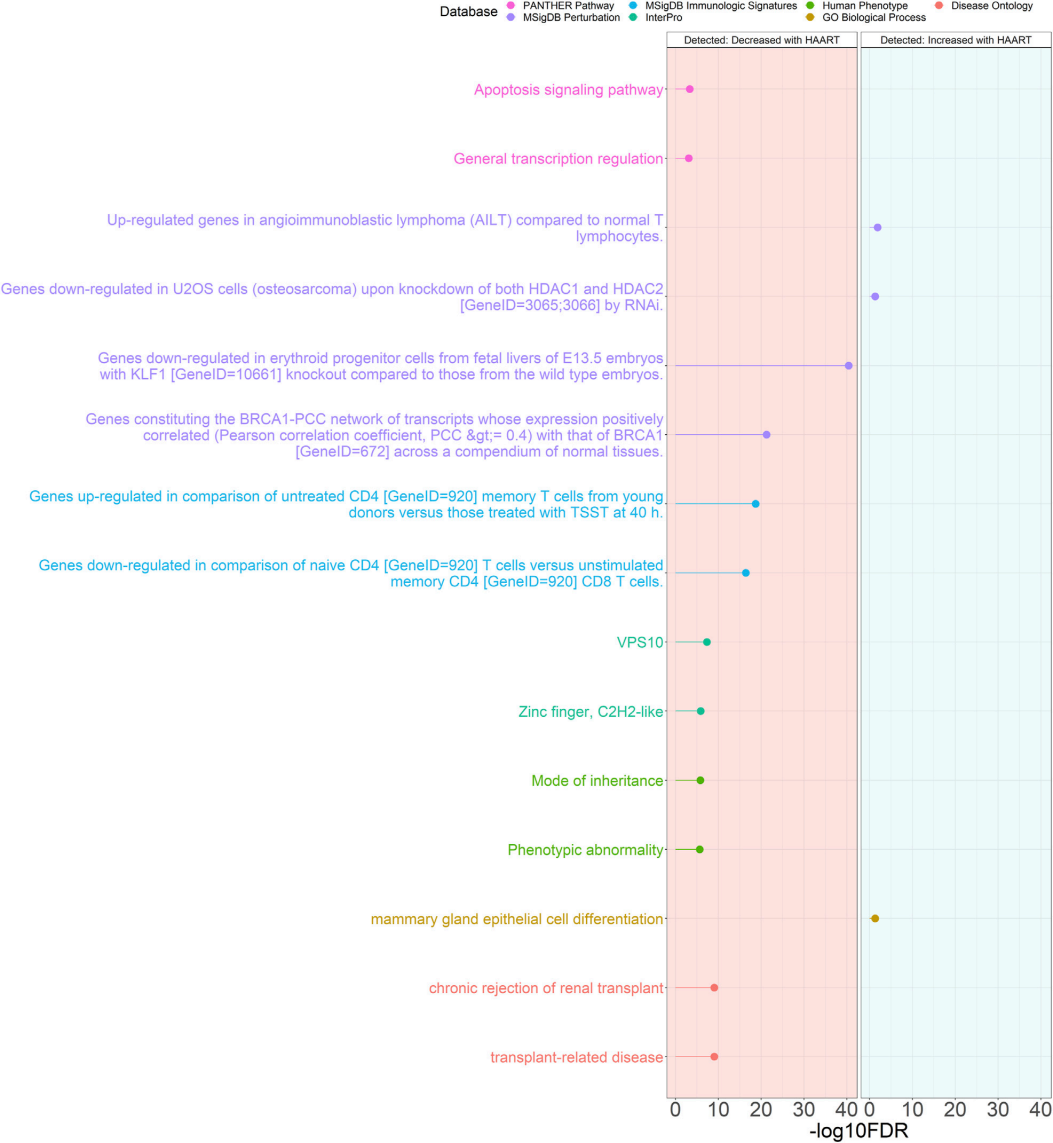
Genomic Regions Enrichment of Annotations Tool for Detected PLWH groups. GREAT results for HAART-related CpGs in Detected PLWH groups. The top 2000 positively-correlated CpGs (increased methylation) and the top 2000 negatively-correlated CpGs (decreased methylation) were taken from the EWAS of HAART initiation to use for the GREAT analysis. The graph with the red background shows the enrichment of the set of negatively-correlated CpGs, while the blue background is the enrichment of the set of positively-correlated CpGs. The significant annotations (*y*-axis) are plotted against the -log10 FDR-adjusted hypergeometric *p*-value (*x*-axis). The ontology databases are grouped by color.

The results of the GREAT functional annotation for the Detected PLWH group are shown in Figure 5. A total of 1,117 annotations passed the significance thresholds (Supplementary Table S4). Out of the 1,114 significant annotations enriched in genes closest to CpGs demonstrating loss of methylation, the most significant annotations were “chronic rejection of renal transplant” (FDR = 8.91 × 10^−10^), “mode of inheritance” (FDR = 1.54 × 10^−6^), “VPS10” (FDR = 4.65 × 10^−8^), “genes upregulated in comparison of untreated CD4 [GeneID = 920] memory T cells from young donors versus those treated with TSST at 40 h” (FDR = 2.03 × 10^−19^), “genes downregulated in erythroid progenitor cells from fetal livers of E13.5 embryos with KLF1 [GeneID = 10661] knockout compared to those from the wild type embryos” (FDR = 3.92 × 10^−41^), and “apoptosis signaling pathway” (FDR = 4.81 × 10^−4^). Only 3 annotations were significantly enriched in genes closest to CpGs demonstrating gain of methylation, consisting of “mammary gland epithelial cell differentiation” (FDR = 4.98 × 10^−2^), “upregulated genes in angioimmunoblastic lymphoma (AILT) compared to normal T lymphocytes” (FDR = 1.41 × 10^−2^), and “genes downregulated in U2OS cells (osteosarcoma) upon knockdown of both HDAC1 and HDAC2 [GeneID = 3065; 3066] by RNAi” (FDR = 4.98 × 10^−2^).

**FIGURE 6:**
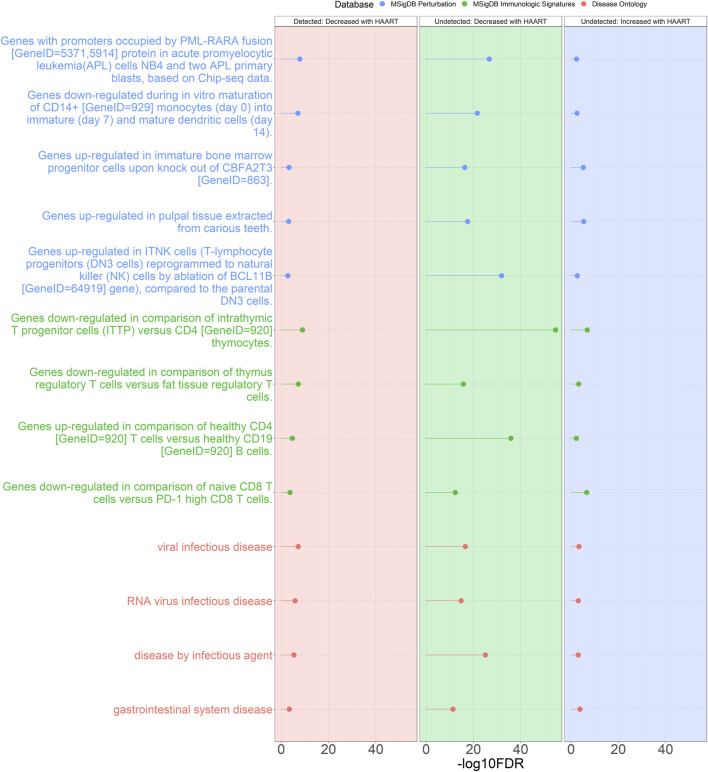
Genomic Regions Enrichment of Annotations Tool for annotations shared between Undetected and Detected PLWH groups. GREAT results for shared annotations between the Undetected and Detected HAART-related CpG GREAT results. Depicted are the Detected group negatively correlated HAART-related CpGs (red background), Undetected group negatively correlated HAART-related CpGs (green background), and Undetected group positively correlated HAART-related CpGs (blue background). The criteria for selecting these annotations were annotations that were shared between at least three sets of GREAT results that were also within the top two most significant results from each set. The significant annotations (*y*-axis) are plotted against the -log10 FDR-adjusted hypergeometric *p*-value (*x*-axis). The ontology databases are grouped by color.

Additionally, many annotations overlapped between the Undetected and Detected groups. The criteria for selection were annotations that were shared between at least three out of the four sets of GREAT results (Undetected Decreased, Undetected Increased, Detected Decreased, Detected Increased) and were also one of the top two most significant annotations for each set of GREAT results (Figure 6). Under these criteria, the Detected Increased GREAT results did not have any overlaps with the other three sets of GREAT annotations.

These shared annotations included “genes downregulated in comparison of intrathymic T progenitor cells (ITTP) versus CD4 [GeneID = 920] thymocytes” (most significant FDR = 1.88 × 10^−55^), “genes upregulated in comparison of healthy CD4 [GeneID = 920] T cells versus healthy CD19 [GeneID = 920] B cells” (most significant FDR = 1.41 × 10^−36^), “genes upregulated in ITNK cells (T-lymphocyte progenitors (DN3 cells) reprogrammed to natural killer (NK) cells by ablation of BCL11B [GeneID = 64919] gene), compared to the parental DN3 cells” (most significant FDR = 1.27 × 10^−32^), “genes with promoters occupied by PML-RARA fusion [GeneID = 5371,5914] protein in acute promyelocytic leukemia (APL) cells NB4 and two APL primary blasts, based on Chip-seq data” (most significant FDR = 1.97 × 10^−27^), and “disease by infectious agent” (most significant FDR = 8.58 × 10^−26^).

### 3.4 Epigenome-wide association studies of potential effects attributable to HAART only

**FIGURE 7:**
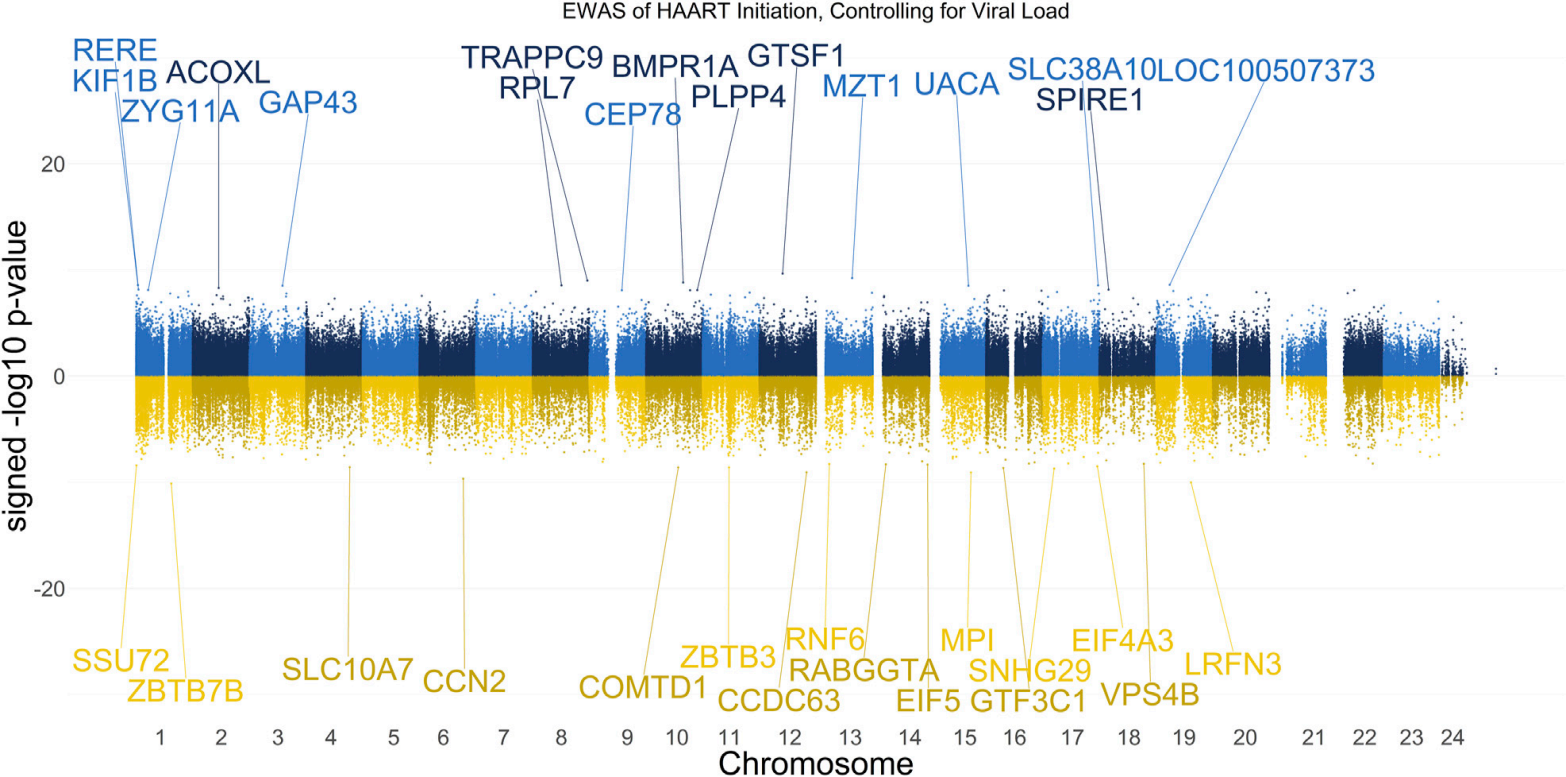
Manhattan Plot of EWAS over the course of HAART Initiation in PLWH, controlling for HIV viral load. Plotted are the HAART-related CpGs in 177 PLWH, controlling for the log10-transformed HIV viral load value of each sample at each visit. Top CpGs are labeled with proximal genes, the *x*-axis represents the location of the CpG on the chromosome, and the *y*-axis represents the signed -log10 *p*-value. The positive y-values (signed -log10 *p*-value) are CpGs that gain methylation with HAART initiation (blue), while the negative y-values are CpGs that lose methylation with HAART initiation (gold).

Prior analyses showed that the mean CpG methylation in Undetected PLWH post-HAART samples exceeded the mean CpG methylation of SN controls at the equivalent visit (Figure 3), potentially indicating HAART-related changes in methylation unrelated to HIV infection. Our next analysis thus focused on CpGs that experienced changes in methylation post-HAART regardless of a PLWH’s change in plasma VL in response to HAART. Therefore, we performed an EWAS using a linear mixed-effect regression model similar to the prior analysis, but included a term to control for the VL (Figure 7). The VL for each sample in this analysis is a continuous variable that represents the log10-transformed number of viral copies per mL at the visit. Ten PLWH were missing VL information at the pre-HAART visit and were therefore removed from both visits, leaving 177 PLWH (354 PLWH samples total) for this analysis.

The results of our VL-controlled HAART EWAS that examines CpGs associated with HAART initiation while adjusting for viral load are shown in Figure 7. A total of 180 CpGs passed the adjusted significance threshold of *p*-value < 1 × 10^−7^ (Supplementary Table S5). Out of the 101 significant CpGs demonstrating loss of methylation from pre-to post-HAART, the most significant CpGs are located in the promoter of ZBTB7B (cg23237634, *p*-value = 7.19 × 10^−11^), the intergenic region downstream of LRFN3 (cg24440088, *p*-value = 9.54 × 10^−11^), the exon of CCN2 (cg14925271, *p*-value = 2.05 × 10^−10^), the promoter of MPI (cg04365443, *p*-value = 8.31 × 10^−10^), and the promoter of CCDC63 (cg00658626, *p*-value = 8.65 × 10^−10^). Out of the 79 significant CpGs demonstrating gain of methylation from pre-to post-HAART, the most significant CpGs are located in the promoter of *GTSF1* (cg18777699, *p*-value = 2.28 × 10^−10^), the intergenic region downstream of *MZT1* (cg19565241, *p*-value = 5.96 × 10^−10^), the intron of *TRAPPC9* (cg21351483, *p*-value = 1.03 × 10^−9^), the intron of *BMPR1A* (cg18525126, *p*-value = 1.50 × 10^−9^), and the exon of *LOC100507373* (cg22791904, *p*-value = 2.49 × 10^−9^).

**FIGURE 8:**
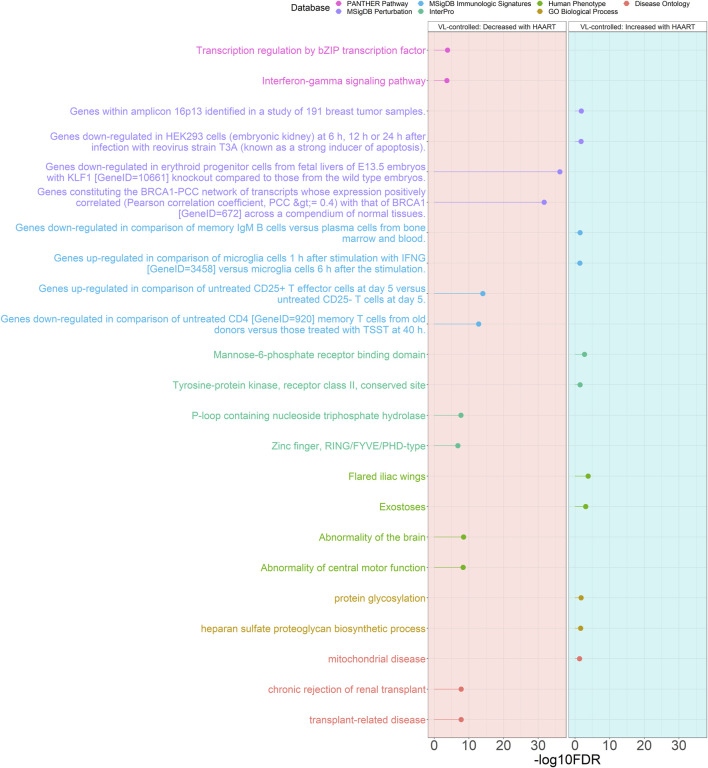
Genomic Regions Enrichment of Annotations Tool for VL-controlled HAART Initiation EWAS CpGs. GREAT results for significant CpGs from VL-controlled HAART-related EWAS in PLWH (*n* = 177). The significant annotations (*y*-axis) are plotted against the -log10 FDR-adjusted hypergeometric *p*-value (*x*-axis). The ontology databases are grouped by color. The graph with the red background is the enrichment of the set of negatively-correlated CpGs (decreased methylation), while the blue background is the enrichment of the set of positively-correlated CpGs (increased methylation).

The most significant CpGs were selected from the VL-controlled HAART EWAS CpGs for GREAT analysis, consisting of the top 2000 positively and top 2000 negatively correlated CpGs. Each set of positively and negatively correlated CpGs were separately annotated using GREAT and the resulting annotations were subjected to a cutoff of an FDR-adjusted *p*-value of < 0.05 and a non-FDR-adjusted hypergeometric *p*-value of < 0.001 (Figure 8). A total of 1,170 annotations passed the significance thresholds (Supplementary Table S6).

Out of the 1,115 significant annotations enriched in genes closest to CpGs demonstrating loss of methylation, the most significant annotations were “chronic rejection of renal transplant” (FDR = 1.71 × 10^−8^), “abnormality of the brain” (FDR = 3.39 × 10^−9^), “p-loop containing nucleoside triphosphate hydrolase” (FDR = 2.02 × 10^−8^), “genes upregulated in comparison of untreated CD25^+^ T effector cells at day 5 versus untreated CD25^−^ T cells at day 5” (FDR = 1.00 × 10^−14^), “genes downregulated in erythroid progenitor cells from fetal livers of E13.5 embryos with KLF1 [GeneID = 10661] knockout compared to those from the wild type embryos” (FDR = 5.48 × 10^−37^), and “transcription regulation by bZIP transcription factor” (FDR = 1.46 × 10^−4^).

Out of the 55 significant annotations enriched in genes closest to CpGs demonstrating gain of methylation, the most significant annotations were “mitochondrial disease” (FDR = 4.91 × 10^−2^), “protein glycosylation” (FDR = 1.75 × 10^−2^), “flared iliac wings” (FDR = 1.60 × 10^−4^), “mannose-6-phosphate receptor binding domain” (FDR = 1.80 × 10^−3^), “genes downregulated in comparison of memory IgM B cells versus plasma cells from bone marrow and blood” (FDR = 3.32 × 10^−2^), and “genes within amplicon 16p13 identified in a study of 191 breast tumor samples” (FDR = 1.48 × 10^−2^).

### 3.5 Equivalent PLWH and SN CpGs that intersect with VL-controlled HAART EWAS

**FIGURE 9:**
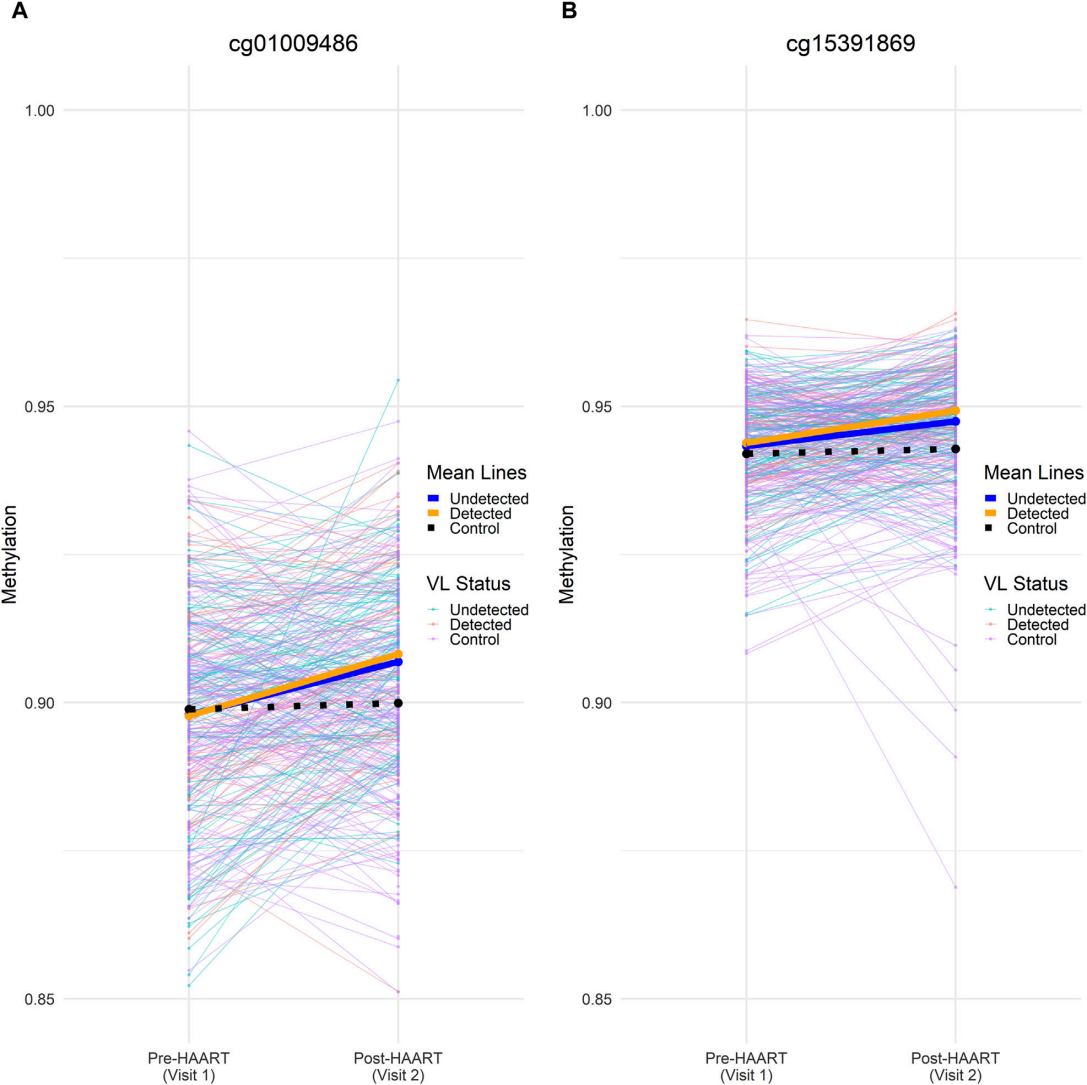
Methylation beta values across visits for two significant CpGs at the intersection of visit 1/pre-HAART equivalence and visit 2/post-HAART differences. Individual (thin lines) and mean (heavy lines) methylation values at visit 1 (pre-HAART in PLWH) and visit 2 (post-HAART in PLWH) are shown for **(A)** cg01009486 and **(B)** cg15391869. Undetected PLWH (blue), detected PLWH (orange), and SN (black) mean lines are shown on top of the mean lines for individual CpGs. Mean methylation was calculated at pre-HAART and post-HAART initiation by averaging the samples across all CpGs. Both CpGs shown here were found to be significantly affected by HAART initiation in the VL-controlled HAART EWAS that consisted of PLWH samples. Both CpGs were also able to reject the null equivalence hypothesis at an FDR-adjusted *p*-value < 0.05 and were unable to reject the null significance hypothesis at an FDR-adjusted *p*-value of < 0.05 in the TOST procedure.

In order to further examine the effects of HAART on methylation while adjusting for viral load, we focused on CpGs that were similar between cases and controls before HAART, but which changed only in cases after HAART initiation. We compared the 177 PLWH (with viral load data available) with 177 matched SN using an equivalence test (“two one-sided *t*-test,” TOST) to find CpGs that were equivalent between these two groups at Visit 1 (pre-HAART). The intersection of this set of CpGs with the set of epigenome-wide significant CpGs (*p* < 1 × 10^−7^) from the VL-controlled HAART EWAS selects for CpGs that were equivalent in methylation level among PLWH and SN controls pre-HAART initiation, but were significantly affected by HAART initiation in the VL-controlled HAART EWAS adjusted for viral load (Figure 9).

Two CpGs were found to pass an epigenome-wide significance level of *p* < 1 × 10^−7^ from the VL-controlled EWAS of HAART as well as being able to reject the null equivalence hypothesis at an FDR-adjusted *p*-value < 0.05 while being unable to reject the null significance hypothesis of zero effect at an FDR-adjusted *p*-value of < 0.05. The two CpGs are located in the intron of *SULT1A1* (cg01009486, *p*-value = 8.82 × 10^−9^) and the intergenic region downstream of *TRERNA1* (cg15391869, *p*-value = 1.23 × 10^−8^).

## 4 Discussion

In this study, we demonstrate that HAART initiation is associated with changes in the mean methylation of CpGs across the genome, affecting a wide variety of CpGs and genes. In CpGs where there are significant differences in mean methylation in PLWH compared with matched SN controls, the mean methylation levels trended towards SN controls after the initiation of HAART. The observed pattern of a persistent but reduced degree of aberrant methylation values in PLWH compared with SN after initiation of HAART is consistent with similar observations in epigenetic clock acceleration measures (Sehl et al., 2020). We further demonstrate that the methylation changes observed from pre-to post-HAART differ when stratifying PLWH for undetected or detected viral load. Of note, we further identified 2 CpGs for which the mean methylation changed significantly away from SN controls and from pre-HAART values in PLWH, suggesting these changes were associated with HAART initiation independent of HIV infection.

We found that HAART initiation has a significant effect on CpGs near genes associated with actin binding. PLEKHG3 is capable of binding newly polymerized actin and thus regulating cell motility and polarity (Nguyen et al., 2016). CAP1 promotes actin filament depolymerization and suppresses spontaneous actin filament polymerization (Bertling et al., 2004). AFAP1 can bind and crosslink actin filaments and may be involved in cytoskeleton remodeling (Snyder et al., 2011). HIV is known to exploit cellular actin networks to promote the spread of infection and compromise the host’s immune system (Ospina Stella and Turville, 2018), potentially indicating that such functions are reflected in the methylome.

The annotations “chronic rejection of renal transplant” and “transplant-related disease” appear at high significance in the GREAT results within the analysis within the Detected group of PLWH, within our VL-controlled analysis. A potential explanation for the prominence of these annotations is that CD4+T cells are targeted by HIV (Balasubramaniam et al., 2019) while also being a major mediator of allograft rejection (Ingulli, 2010). Prior studies have indicated that DNA methylation may be a potential biomarker for monitoring kidney transplants, including identifying or predicting patients that may be at risk for rejection (Cristoferi et al., 2022). Additionally, it has been shown that immune-related methylation changes occur in acute rejection-induced allograft dysfunction in mice (Zhu et al., 2021).

The annotation “Genes downregulated in erythroid progenitor cells from fetal livers of E13.5 embryos with KLF1 [GeneID = 10661] knockout compared to those from the wild type embryos” is highly significant in our analyses examining HAART-related CpGs within the Detected group as well as the VL-controlled analyses. KLF1 mutations may be associated with elevated levels of hemoglobin A2 (Srivorakun et al., 2020). Likewise, some antiretroviral therapy drugs may elevate hemoglobin A2 in HIV-positive adults (Bhagat et al., 2015), potentially providing an explanation for the significance of this annotation.

One of the pressing questions of HAART is whether antiretroviral therapy itself alters the epigenome. Here we demonstrate that HAART initiation is associated with significant methylation changes in CpGs even when controlling for viral load. Interestingly, the most significantly negatively correlated VL-controlled HAART CpG (cg23237634, *p*-value = 7.19 × 10^−11^) is located in the promoter of ZBTB7B, a gene that plays a role in CD4^+^ T cell differentiation. Expression of this gene may be elevated in “elite controllers” (individuals able to suppress viral loads and remain asymptomatic without antiretroviral therapy) compared to individuals who have not received antiretroviral therapy (De Arcos-Jiménez et al., 2020).

After filtering the VL-controlled HAART EWAS for CpGs that reach genome-wide significance (*p* < 1 × 10^−7^) and are equivalent under the TOST procedure, two CpGs remain. They are located 1) in the intron of *SULT1A1* (cg01009486, *p*-value = 8.82 × 10^−9^) and 2) the intergenic region downstream of *TRERNA1* (cg15391869, *p*-value = 1.23 × 10^−8^). Both CpGs gain methylation post-HAART initiation. Sulfotransferase family 1A member 1 (SULT1A1) promotes HIV-1 infection of primary human monocyte-derived macrophages by regulating retroviral reverse transcription (Swann et al., 2016). Furthermore, increased copy counts of SULT1A1 may also be associated with reduced plasma concentrations of efavirenz, which is a common component of HAART (Chamnanphon et al., 2021). Translational regulatory lncRNA 1 (TRERNA1) is a long non-coding RNA that is associated with increasing diffuse large B cell lymphoma proliferation when *N* ^*6*^-methyladenosine methylation levels are decreased in the transcript (Song et al., 2022). TRERNA1 is upregulated in gastric cancer with a role in promoting metastasis (Wu et al., 2018). One of the major regulators of TRERNA1 is TFAP4, which is also known to bind to long terminal repeats in HIV-1 to repress viral gene expression (Imai and Okamoto, 2006).

One limitation of this study is the diversity and changing nature of drug regimens the participants were prescribed. Participants of this study were using older drug regimens that may not accurately reflect results obtained from newer drug regimens. It would be important to repeat this type of analysis on study participants exposed only to current therapy regimens.

Overall, we find that HAART initiation alters CpG methylation in genes associated with immune responses and HIV infection, with post-HAART CpG methylation in PLWH trending toward SN controls. Because HAART initiation may also alter CpG methylation independent of HIV infection, further studies are needed to disentangle the effects of HAART itself on the methylome that are not related to controlling HIV viral load.

## Data Availability

The data analyzed in this study is subject to the following licenses/restrictions: The raw Infinium MethylationEPIC BeadChip methylation data that support the findings reported in this study cannot be deposited in a public repository at this time because of the policies of the MACS/WIHS Combined Cohort Study (MWCCS) from which they were generated. Per MWCCS policies, these raw data will be released via a concept sheet approval process (https://statepi.jhsph.edu/mwccs/work-with-us/) once the original aims of our approved study are complete. All analytic data utilized in this paper (necessary de-identified demographic or descriptive data), have been deposited with the MWCCS, and are available upon reasonable request via the MWCCS concept sheet approval process (https://statepi.jhsph.edu/mwccs/work-with-us/). All data related to this paper was obtained under MWCCS concept sheet C15039. Requests to access these datasets should be directed to https://statepi.jhsph.edu/mwccs/work-with-us/.
